# A Novel Cell-Penetrating Antibody Fragment Inhibits the DNA Repair Protein RAD51

**DOI:** 10.1038/s41598-019-47600-y

**Published:** 2019-08-02

**Authors:** Landon Pastushok, Yongpeng Fu, Leo Lin, Yu Luo, John F. DeCoteau, Ken Lee, C. Ronald Geyer

**Affiliations:** 1 Department of Pathology and Lab Medicine, University of Saskatchewan, Saskatoon, Canada; 2 Department of Biochemistry, University of Saskatchewan, Saskatoon, Canada; 3 Advanced Diagnostics Research Lab, Saskatchewan Cancer Agency, Saskatoon, Canada; 4 iProgen Biotech Inc., Burnaby, Canada

**Keywords:** Protein delivery, Antibody fragment therapy

## Abstract

DNA damaging chemotherapies are successful in cancer therapy, however, the damage can be reversed by DNA repair mechanisms that may be up-regulated in cancer cells. We hypothesized that inhibiting RAD51, a protein involved in homologous recombination DNA repair, would block DNA repair and restore the effectiveness of DNA damaging chemotherapy. We used phage-display to generate a novel synthetic antibody fragment that bound human RAD51 with high affinity (K_D_ = 8.1 nM) and inhibited RAD51 ssDNA binding *in vitro*. As RAD51 is an intracellular target, we created a corresponding intrabody fragment that caused a strong growth inhibitory phenotype on human cells in culture. We then used a novel cell-penetrating peptide “iPTD” fusion to generate a therapeutically relevant antibody fragment that effectively entered living cells and enhanced the cell-killing effect of a DNA alkylating agent. The iPTD may be similarly useful as a cell-penetrating peptide for other antibody fragments and open the door to numerous intracellular targets previously off-limits in living cells.

## Introduction

Chemotherapy is a predominant strategy for treating cancer. With the exception of targeted therapies, most chemotherapy works on the premise that drugs causing DNA damage kill rapidly dividing cancer cells better than their normal counterparts. Unfortunately, chemotherapy often fails to eliminate cancer cells due to innate or acquired drug resistance mechanisms that prevent drug activity^1,2^. Further, normal DNA repair processes can counteract DNA damage-based chemotherapies^3^, and this mechanism of chemo-resistance is further compounded in cells that have misregulated DNA repair^4,5^.

Homologous recombination (HR) is a well-conserved and fundamentally important DNA repair pathway that uses homologous undamaged sister chromatids as an error-free template to restore genetic information. HR is typically responsible for double-strand break (DSB) repair, however, the HR machinery is also used for a “last-resort” process to overcome a wide variety of potentially lethal DNA modifications that may be encountered by the DNA replication fork. Various DNA lesions from base modifications and bulky adducts to intra- and inter-strand cross-links may all lead to single-ended DSBs, which if left un-repaired can lead to cell death. This is in contrast to other major DNA repair mechanisms, such as base excision repair, mismatch repair, and non-homologous end joining, which have a comparatively narrow spectrum of target lesions. The broad functions of HR proteins is highlighted by the participation of HR mechanisms in the repair of all major toxic lesions induced by cancer treatments with the exception of anti-metabolites^3^. HR proteins are therefore central to repairing chemotherapy induced DNA damage.

RAD51 is an essential^6^ HR recombinase conserved in nature^7^ that conventionally functions in two important homology-directed DNA repair steps. First, following resection of DSB lesions, RAD51 uses DNA binding^8^, ATP-binding, and ATPase activities^9,10^ to form a nucleoprotein filament. Next, the DNA-RAD51 nucleofilament promotes strand invasion^11,12^ of a homologous DNA duplex to form the characteristic D-loop DNA structure necessary for recombination. These activities are carried out in conjunction with and mediated by several protein-protein interactions^13–16^. Beyond this classic HR role, RAD51 is also necessary for replication fork reversal and chromatin remodeling^17^ in S-phase to address a variety of damage-induced lesions encountered during DNA replication^18,19^. Further, RAD51 is involved in replication fork maintenance by protecting nascent DNA strands from nuclease degradation during replication^20–23^.

The central activities of RAD51 in DNA repair and replication point toward a role in cancer and cancer therapy chemoresistance. Indeed, *RAD51* overexpression is observed in immortal human cancer cell lines^24,25^ and in breast cancer^26^, prostate cancer^27^, pancreatic cancer^28^, non-small cell lung carcinoma^29^, and leukemia primary cancer cells^30^. In addition, RAD51 hyperactivity is thought to contribute fundamentally to the genesis of genome instability and cancer, and *RAD51* overexpression leading to hyper-recombination is implicated in malignant transformation^31,32^. These associated phenotypes are therapeutically relevant, as *RAD51* overexpression increases cellular resistance to radiation and chemotherapeutic drugs^33–35^. Additional connections to cancer have placed RAD51 as a possible biomarker^36^ and prognostic indicator^37^. Taken together, RAD51 is a potential therapeutic target that may be particularly effective for patients undergoing chemotherapy.

Several early studies showed success in inhibiting RAD51 for cancer treatments. Depletion of RAD51 by antisense RNA attenuates radiotherapy resistance^38,39^ and intensifies killing of immortal HeLa cells by cisplatin^40^. Similarly, targeted *RAD51* inhibition using ribozyme treatments increases radiosensitivity^41^. These studies laid the foundation for targeting RAD51 with several small-molecule inhibitors. Those most extensively studied include DIDS^42,43^, B02^44,45^, IBR2^46^, Halenaquinone^47^, and RI-1/RI-2^48,49^. Together they inhibit various RAD51 activities, including homologous strand pairing and exchange, D-loop formation, ssDNA binding, and dsDNA binding^50^. Despite limited success in potentiating chemotherapeutic agents, most of these small molecules are limited by poor specificity, instability, and cellular toxicity leading to side-effects in patients. As a result, most RAD51 small molecule inhibitors to date have been limited to *in vitro* and research purposes^51^.

With shortcomings for small-molecule chemotherapeutics in treating RAD51-associated cancer, we hypothesized that an entirely different class of drugs might be successful. Therapeutic antibodies are prominent anti-cancer drugs^52^ that can offer several advantages over small-molecules, including tighter target binding, improved specificity, and longer *in vivo* half-lives^53^. However, due to their large size, antibodies and antibody fragments do not effectively enter living cells, and are normally limited to extracellular and cell-surface targets^54^. In order to inhibit RAD51 with an antibody, we fused a cell-penetration peptide (CPP) called “iPTD” to a RAD51 inhibitory antigen-binding fragment (Fab). The resulting Fab-iPTD was able to penetrate living cells and enhance the cell-killing activity of a DNA alkylating agent.

## Results

### Generation of a human antigen-binding fragment (Fab) against human RAD51

**Figure 1:**
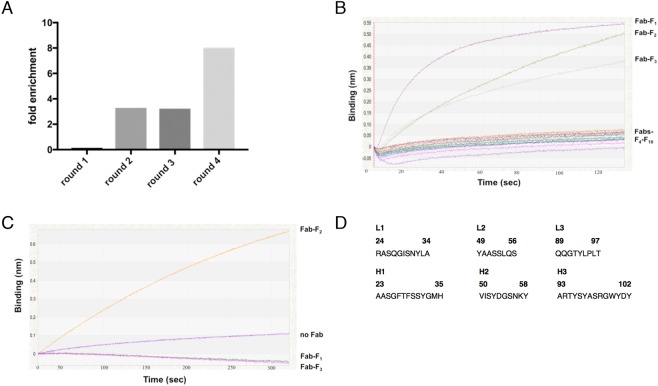
Selection of a Fab binding to RAD51. (**A**) Four rounds of phage display selection were performed and fold enrichment was calculated as eluted phage from target wells containing immobilized RAD51 divided by BSA negative control wells. (**B**) *E*. *coli* lysates from 18 random clones over-expressing Fab were sampled in parallel using OctetRED384 biolayer interferometry. The presence of expressed Fab is detected by binding to Protein A biosensors which causes an increase in optical thickness or binding (nm) over time. (**C**) Fabs immobilized to protein L biosensors are transferred to wells containing 500 nM RAD51 and the resulting association curves are shown. (**D**) Fab-F2 CDR sequences. The Kabat scheme was used for numbering amino acids and the CDR regions shown were defined according to North *et al*.^91^.

Recombinant human RAD51 was purified from *E*. *coli* (Fig. S3) and used as the target for phage display selection of Fab fragments (Fig. 1). We used a novel human IgG_1_ synthetic Fab phage library containing >1 × 10^10^ members, referred to as Library S^55^. Library S was designed with canonical CDRs to structurally complement randomized CDRL3 and CDRH3^55^. Phage display selections were performed against surface-immobilized recombinant RAD51 and a modest enrichment of target binding phage over negative BSA controls was observed after four rounds of selection (Fig. 1A). The pool of Fab-encoding sequences from the fourth round of selection was sub-cloned from phage for small-scale Fab expression in *E*. *coli*. Eighteen Fab lysates were screened for Fab expression through successful loading (i.e. immobilization) onto Protein A biosensors using biolayer interferometry (BLI). Of three lysates with loaded Fab (Fig. 1B), only one subsequently bound RAD51 at a 500 nM screening concentration (Fig. 1C). This clone, Fab-F2, was sequenced (Fig. 1D) and purified to near homogeneity (Fig. S1).

### Kinetics and specificity of Fab-F2 binding to RAD51

**Figure 2:**
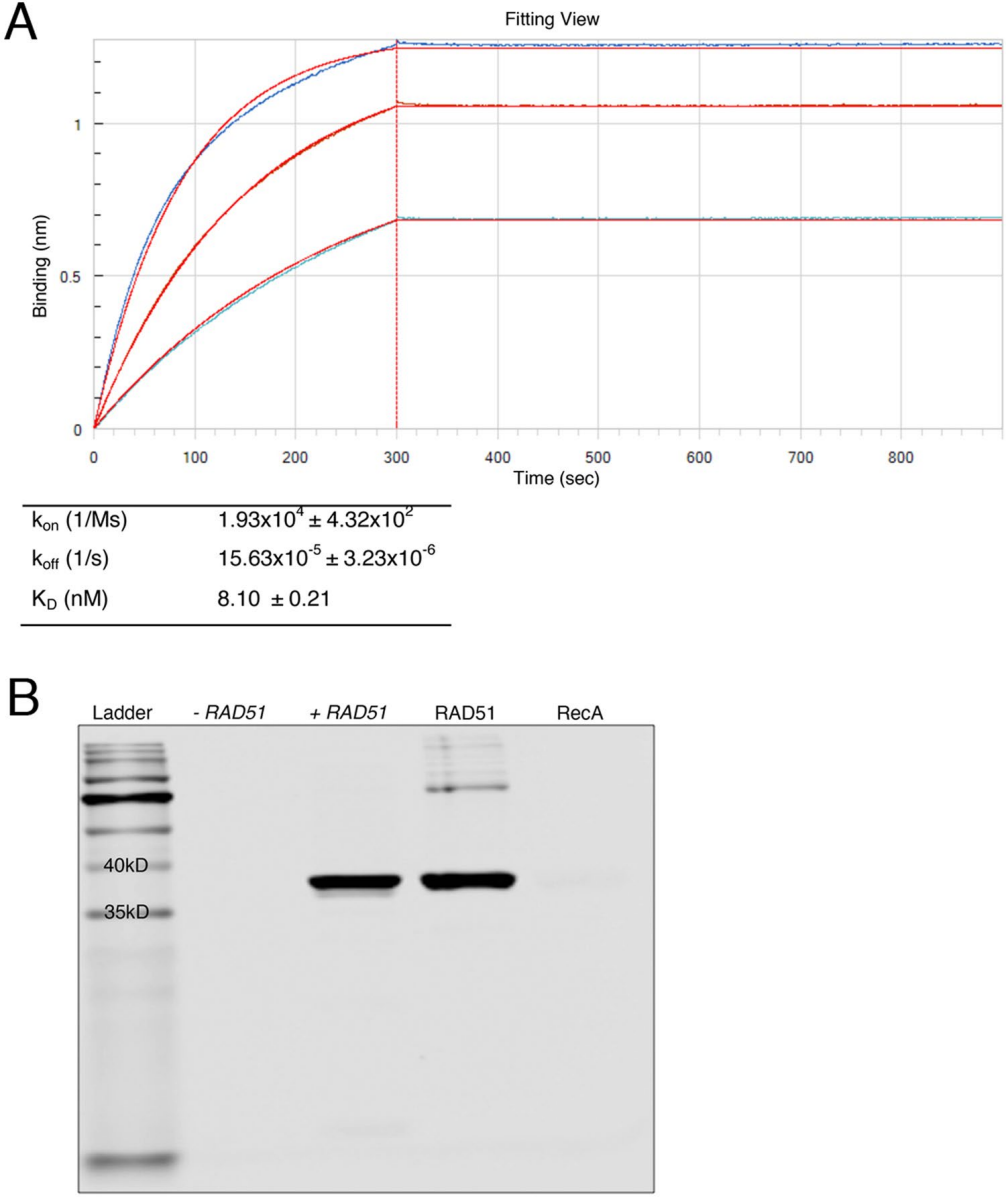
Kinetics and specificity of Fab-F2. (**A**) Kinetic parameters for the binding of Fab-F2 to purified RAD51 were determined using biolayer interferometry. Equal amounts of Fab-F2 were immobilized on Protein L biosensors which were immersed in parallel to a range of RAD51 concentrations and then buffer alone to yield association and dissociation curves (blue traces), respectively. Global curve-fitting employing a 1:1 Langmuir binding model (red traces) using reference-well subtracted data was used to determine the kinetic values in the table. (**B**) A Western blot using IRDye 800CW-labeled Fab-F2 was used to detect expression of plasmid-based *RAD51* from E *coli* lysates with (+) or without (−) induction by IPTG. Equal amounts of purified RAD51 and the homologue RecA were used to verify size and specificity, respectively. Minor aggregation of purified RAD51 and/or RAD51-DNA impurities was observed (upper bands). The lower ladder band indicates the gel dye-front.

BLI was used to determine kinetics of Fab-F2 binding to RAD51 (Fig. 2A). Protein L biosensors were used to immobilize Fab-F2 and three concentrations of RAD51 were used to measure the association and dissociation phases of the interaction. Binding curves were fit globally to a 1:1 binding model, which exhibited a tight fit and yielded an on-rate (k_on_) of 1.93 × 10^4^ 1/Ms ± 4.32 × 10^2^, an off-rate (k_off_)of 15.63 × 10^−5^ 1/s ± 3.23 × 10^−6^, and a dissociation constant (K_D_) of 8.10 nM ± 0.21.

*E*. *coli* RecA is a structural and functional homologue of RAD51 that shares 51% sequence similarity over its core-domain and a conserved homologous recombination function in DNA strand pairing and exchange^56,57^. To test binding specificity, Western analysis using Fab-F2 against equal amounts of purified RAD51 and RecA was performed. Fab-F2 was able to detect RAD51 but not RecA (Fig. 2B). Further specificity was observed through the detection of a single band in *E*. *coli* whole cell lysates containing plasmid-expressed RAD51 versus the control expression plasmid (Fig. 2B).

### Fab-F2 inhibits RAD51 DNA binding but not ATPase activity *in vitro*

**Figure 3:**
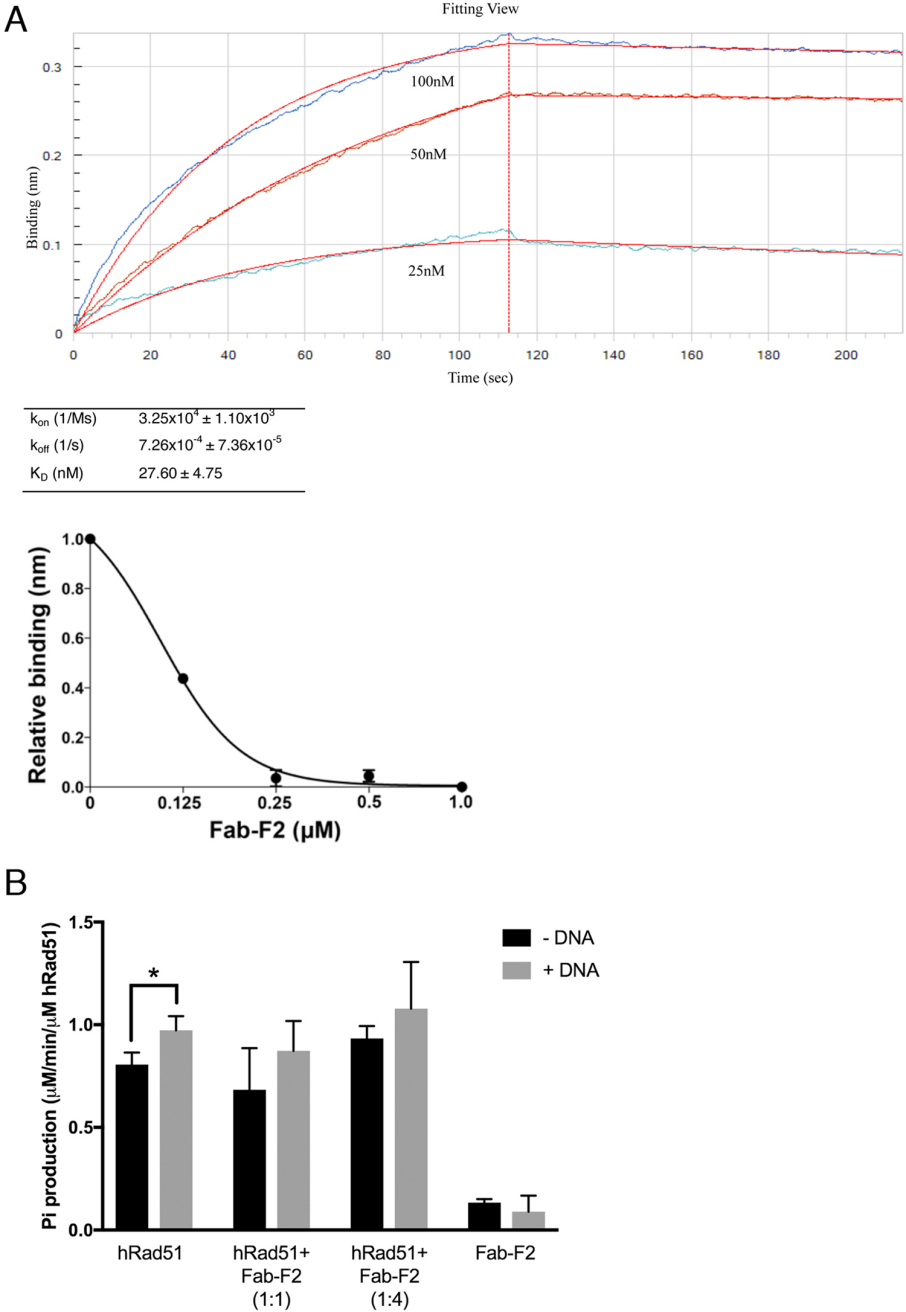
Inhibition of RAD51 activities *in vitro* by Fab-F2. (**A**) Biolayer interferometry was used to assay the inhibition of RAD51 ssDNA binding by Fab-F2. First, the kinetics of RAD51 binding to DNA was determined (upper panel) using streptavidin-immobilized 5′-biotinylated oligo(dT)_36_ biosensors which were immersed in parallel to a range of RAD51 concentrations and then buffer alone to yield association and dissociation curves (blue traces), respectively. Global curve-fitting employing a 1:1 Langmuir binding model (red traces) using reference-well subtracted data was used to determine the kinetic values in the table. In the lower panel, the experiment was repeated using 0.5 μM RAD51 so that maximal binding at equilibrium in the absence of Fab-F2 (expressed as 1.0) could be plotted relative to binding in the presence of a range of Fab-F2 concentrations. Error bars indicate standard deviation from three independent experiments. (**B**) The effect of Fab-F2 on ATP hydrolysis by RAD51 was measured with a malachite green assay that enables spectrophotometric detection of free phosphates. The free inorganic phosphates produced by RAD51 (μM/min/μM RAD51) from ATP were determined in reactions containing RAD51 or Fab-F2 alone and in combination at the indicated ratios in the presence and absence of ssDNA. Error bars represent standard deviation from at least three independent measurements. *p-value 0.034.

RAD51 posses several activities for DNA repair. In its role in HR, RAD51 binding to ssDNA is an integral part of DNA nucleoprotein filament formation, which is essential for the creation of a pre-synaptic filament/complex^58–60^. ssDNA binding by RAD51 is also a requirement for its role in protecting the DNA replication fork^22^. To test Fab-F2 inhibition of RAD51 ssDNA binding, we established a BLI DNA-binding assay. We first determined the K_D_ of the RAD51-ssDNA interaction by immobilizing 5′-biotinylated poly-deoxythymidine (oligo(dT)_36_) onto a streptavidin biosensor and measuring RAD51 binding (Fig. 3A). The ssDNA-RAD51 K_D_ was 27.60 ± 4.75 nM, which is similar to the previously reported K_D_ for the interaction of RAD51 with oligo(dT)_50_^61^. To measure Fab-F2 inhibition of the ssDNA-RAD51 interaction, we repeated BLI in a similar manner, except with pre-incubation of 500 nM RAD51 with various concentrations of Fab-F2. This enabled measurement of RAD51 ssDNA binding inhibition by subtraction of binding signals without Fab-F2. Using this approach, an IC_50_ for the Fab-F2 was calculated to be less than 125 nM (Fig. 3A).

RAD51 binds ATP to promote its own stable nucleofilament formation^10,62^. Following strand invasion and homology searching functions, RAD51 then hydrolyzes ATP through its ATPase domain, causing its release from the synapse^11,12,63^. To investigate whether Fab-F2 inhibits RAD51 ATPase activity, we monitored the release of inorganic phosphate by ATP hydrolysis over time using a malachite green assay^64^. The lower than anticipated yet statistically significant stimulation of ATPase activity in the presence of ssDNA may have been due to possible residual nucleic acid in the RAD51 preparations, as indicated in Fig. 2B. Nonetheless, the RAD51 ATPase activity was not inhibited by Fab-F2 to any extent in the presence or absence of ssDNA (Fig. 3B).

### Intracellular expression of a Fab-F2 scFv fragment inhibits HEK293T cell growth

To determine the potential for Fab-F2 to affect RAD51 function in cells, we sub-cloned the light and heavy chain variable regions of Fab-F2 as a single chain variable fragment (scFv) that could be expressed inside cells. An scFv is encoded by a single gene that brings together the antigen-binding variable heavy and light chains with a flexible poly-linker^65,66^. To improve intracellular function, the scFv construct was fused to a fragment crystallizable (Fc) domain to enhance stability and a nuclear localization signal to promote nuclear uptake^67^. A cell import tag (iPTD, see below) was also added to streamline with prospective recombinant Fab-F2-iPTD. The construct was then cloned into the mammalian constitutive expression plasmid pcDNA and transiently transfected into HEK293T cells. scFv-Fc expression was confirmed by Western analysis of cell lysates (Fig. S2).

To test scFv-F2-Fc-iPTD on RAD51 DNA repair function in cells, we used the alkylating agent methylmethane sulfonate (MMS) to induce DNA damage and replication fork impairment^68,69^. The mutation of genes involved in homologous recombination DNA repair, such as *RAD51*, cause sensitivity to the alkylating agent MMS^70^. In addition, we expected MMS-induced replication fork impairment would impinge upon the role of RAD51 in HR-independent DNA replication fork fidelity^22^. Lastly, MMS induces RAD51 foci formation^71^ and is relevant to our overall goal in treating cancer with Fab-F2 because alkylating agents are commonly used in chemotherapy^3^.

**Figure 4:**
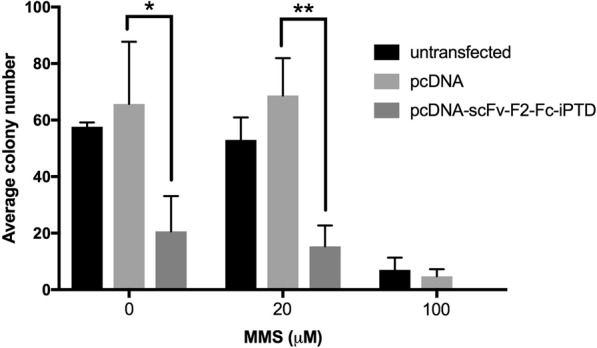
Cells in culture are sensitive to an scFv-Fc intracellular antibody based on Fab-F2. A clonogenic assay was used to test the effect of an intracellular-expressed Fab-F2 derivative. HEK293T cells were transiently transfected with a pcDNA-*SCFV-F2-FC-IPTD* expression plasmid and exposed to methyl methanesulfonate (MMS). Cells were cultured for 7 days post MMS treatment and colonies were stained with 0.3% crystal violent and enumerated by light microscopy. Error bars indicate standard deviation from three independent measurements. *p-value = 0.037 **p-value = 0.0036.

We used a clonogenic survival assay to test transiently transfected HEK293T cells for colony growth as quantified by light microscopy^72^. Cells were seeded at 200 per well and treated with MMS the next day. Following culture for seven days, cells were then stained in order to count viable colony growth as a measure of tolerance to MMS. The pcDNA-SCFV-F2-FC-IPTD construct had a strong effect in preventing HEK293T cells to form colonies, independent of MMS treatment (Fig. 4).

### Fusion of a novel intracellular protein delivery domain, iPTD, with Fab-F2 does not block RAD51 binding

We fused a novel intracellular protein delivery domain iPTD to Fab-F2 for cell internalization. The iPTD (Patent No. WO 2014005219 A1) consists of a 35 amino acid sequence (MALGPCMLLLLLLLGLRLPGVWAPPRRRRRRRRR) that enhances interaction with the cell membrane and incorporates features from each major CPP class: cationic, hydrophobic, and amphipathic^73^. A design feature of the iPTD is enhanced retrograde transport that, similar to immunotoxins and intracellular pathogens, is an efficient and common mechanim relevant to almost any cell type^74,75^. The iPTD also contains a cleavage inhibition sequence (CIS) that enables retention of iPTD-fused cargo with retrotranslocon post cell membrane interactions to facilitate internalization

**Figure 5:**
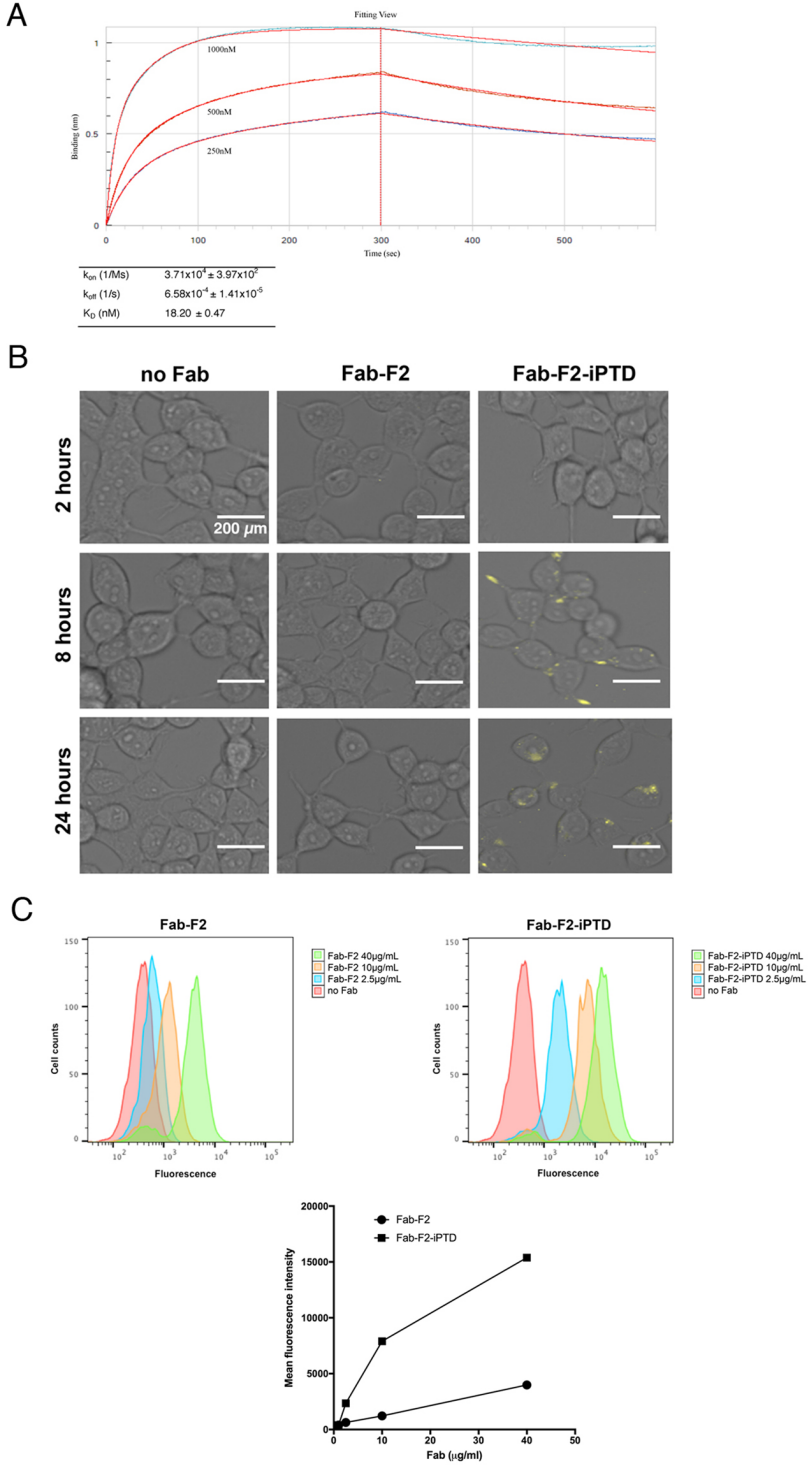
A cell-penetrating Fab-F2-iPTD binds to RAD51. (**A**) Kinetic parameters for the binding of Fab-F2-iPTD to purified RAD51 were determined using biolayer interferometry. Equal amounts of Fab-F2-iPTD were immobilized on Protein L biosensors which were immersed in parallel to a range of RAD51 concentrations and then buffer alone to yield association and dissociation curves (blue traces), respectively. Global curve-fitting employing a 1:1 Langmuir binding model (red traces) using reference-well subtracted data was used to determine the kinetic values in the table. (**B**) HEK293T cells were incubated with 40 µM 800CW-labeled Fab (yellow) for the indicated time points and fluorescent microscopy was used to visualize cellular localization. Image contrast and brightness adjustments were performed equally across all panels. Bars indicate 200 µm. (**C**) Flow cytometry was used to quantify relative Fab internalization. The mean fluorescence intensity was measured for HEK293T cells treated with 800CW-labeled Fab for 24 hours (upper panels) and then plotted for comparison (bottom panel). Error bars, while not visible, were added to indicate standard deviation from three independent measurements.

The iPTD-encoding sequence was cloned in-frame to the heavy chain C-terminus of Fab-F2. This construct, Fab-F2-iPTD, was expressed and purified from *E*. *coli* with yield and purity similar to Fab-F2 (Fig. S1). To verify that the iPTD did not interfere with RAD51 binding, we determined kinetics of Fab-F2-iPTD binding to RAD51. The iPTD had a modest effect on the Fab-F2 interaction with RAD51, with on- and off-rates lowered by approximately 2-fold (Fig. 5A vs. Figure 2A). The K_D_ for Fab-F2-iPTD was 18.20 nM as compared to 8.10 nM for Fab-F2.

### Fab-F2-iPTD exhibits enhanced internalization into HEK293T cells

To compare the internalization of Fab-F2-iPTD versus Fab-F2 in HEK293T cells, we labeled Fab-F2 and Fab-F2-iPTD with the near-infrared dye IRDye^®^800CW, and analyzed cellular localization by fluorescent microscopy. A fluorescent signal could be observed for Fab-F2-iPTD after only eight hours following addition to the cell culture media, with predominant staining at the plasma membrane (Figs 5B and S4). After 24 hours, Fab-F2-iPTD entered cells and exhibited intracellular foci with little to no staining at the plasma membrane. No corresponding fluorescent signal for Fab-F2 was observed. To quantify the relative uptake of Fab-F2-iPTD and Fab-F2, HEK293T cells treated with Fab at the 24-hour time-point were measured by flow cytometry to detect cell-bound fluorescence. Over all concentrations, cells treated with Fab-F2-iPTD exhibited peak shifts greater than cells treated with Fab-F2 (Fig. 5C). The difference was most obvious in a plot of mean fluorescence intensity versus concentration (Fig. 5C), where the Fab-F2-iPTD signal sharply increases at lower concentrations. In contrast, Fab-F2 fluorescence increased linearly in a manner typical of background binding and/or non-specific uptake.

### Fab-F2-iPTD increases the sensitivity of HEK293T cells to MMS

**Figure 6:**
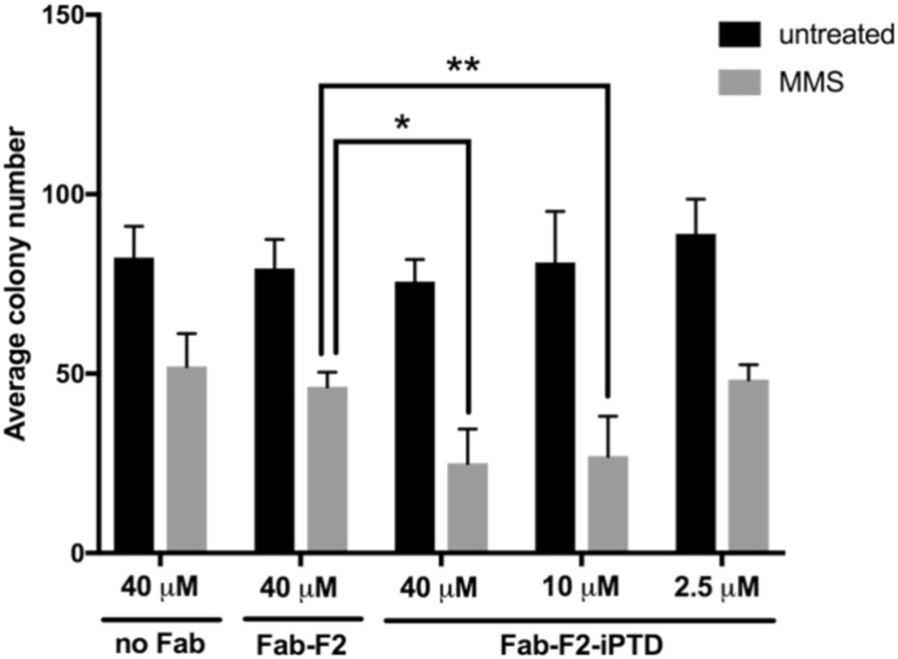
Fab-F2-iPTD increases cell sensitivity to MMS. A clonogenic assay was used to test the effect of adding purified Fab-F2 or -F2-iPTD protein to HEK293T cells treated with MMS at the indicated concentrations. Cells were cultured for seven days post treatment and colonies were then stained with 0.3% crystal violent and enumerated by light microscopy. Error bars represent standard deviation from three independent measurements. No statistical difference exists between untreated samples. *p-value = 0.023 **p-value = 0.042.

We tested Fab-F2-iPTD intracellular function by measuring its ability to enhance the activity of MMS in the clonogenic survival assay. HEK293T cells were treated with purified Fab-F2 or Fab-F2-iPTD in the presence and absence of MMS, and the resulting cell colonies were counted after 7 days. We observed no direct inhibition of clonogenicity for either Fab treatment lacking MMS. However, following MMS treatment, cells exposed to Fab-F2-iPTD at 40 μM and 10 μM caused a significant reduction in colony formation in comparison to Fab-F2 at 40 μM, which had no measurable effect on MMS-induced cell death (Fig. 6).

## Materials and Methods

### Plasmids

The coding sequence of *Homo sapiens* RAD51 was a gift from Richard Fishel at Ohio State University. The coding sequence for residues 21–339 of RAD51 was inserted between the NcoI and XhoI sites of plasmid pET28a (Novagen). The expressed RAD51 protein contained 8 extra amino acid residues (Leu-Glu-His-His-His-His-His-His) at the C-terminus.

To create pCW-Fab-F2 and -iPTD, Fab fragments were PCR amplified from phagemid clones using primers TGS157 (5′-TCCAGATGACCCAGTCCCCGAGCTCCCTG) and TGS160 (5′-CAAATCTTGTGACAAAACTCACACGGGTGGTTCGCACCACCACCACCACCACTGAG). The Fab DNA sequences were cloned by Gibson Assembly^76^ into a modified pCW-LIC (Addgene plasmid #26098) plasmid containing the 5′ portion of the Fab light chain followed by a SacI restriction site, and a 3′ portion containing an XhoI restriction site followed by a poly-His-encoding sequence or a poly-His-encoding sequence preceded by the iPTD encoding sequence (5′-ATGGCC TTGGGCCCTTGCATGTTGTTGTTGTTGTTGTTGTTGGGTTTGCGCCTGCCGGGTGTTTGGGCGCCGCCGCGTCGCCGCCGCCGTCGTCGCCGTCGT).

The gene sequence encoding scFv-F2-iPTD was synthesized in a pUC57 plasmid and subcloned directly (via restriction digestion with KpnI and BamHI) from pUC57 and ligated into the KpnI-BamHI linearized vector of an antibody-expressing pcDNA3.1(+) plasmid containing the Fc-NLS domain. The NLS domain is from the nuclear localization sequence (EGMLANLVEQNISVRRRQGVSIGRLHKQRKPDRRKRSRPYKAKRQ) at the C-terminal end of ubiquitin carboxyl-terminal hydrolase BAP1.

### RAD51 purification

*E*. *coli* Rosetta(DE3) (Novagen) cultures harbouring pET28a-RAD51 were grown at 37 °C until an OD_600nm_ of 1.2, and were then induced with 0.25 mM IPTG for four hours. Cells were harvested by centrifugation and pellets were resuspended in binding buffer (20 mM sodium phosphate, 0.5 M NaCl, 25 mM imidazole at pH 7.4). Following sonication lysates were clarified by centrifugation at 12,000 g for 15 minutes. RAD51 was precipitated in a final concentration of 0.35 g/mL ammonium sulfate. Following centrifugation at 12,000 g for 15 minutes, the protein pellet was dissolved in binding buffer and passed through a DE52 anion exchange column (Sigma-Aldrich) to remove DNA. A HiTrap Ni-chelating column (GE Healthcare) was used as a final purification step per manufacturer’s instructions and eluted with 500 mM imidazole. Fractions containing RAD51 protein were dialyzed with PBS. Protein concentrations were determined using Pierce^TM^ BCA Protein Assay Kit.

### Phage display

Phage display selection with synthetic Fab Library S as described in^55^ was performed by immobilizing 5 μg/mL of purified RAD51 on Nunc MaxiSorp Immunoplates, and 10^13^ total phages each selection round were incubated with RAD51 for 2 hours. Unbound phages were removed with eight washes of PBS and target-bound phages were eluted with acid. Enrichment of target-binding phages was calculated each round by titering the number of phage eluted from RAD51 wells divided by the BSA negative control wells performed in parallel.

### Fab purification

Fabs were purified as described in^55^.

### Small-scale lysate preparation

Soluble protein was extracted from small-scale *E*. *coli* cultures (<5 mL) using B-PER Bacterial Protein Extraction Reagent (ThermoScientific) per manufacturer’s instructions employing a 10 minute room temperature incubation with gentle rotation. The lysate supernatant was separated from insoluble material by centrifugation at 12,000 g for 5 minutes at 4 °C.

### Western blot

HEK293T whole-cell lysates were prepared with SDS sample buffer followed by incubation at 95 °C for 10 minutes. Proteins were separated with 12% SDS-PAGE and blotted onto nitrocellulose. Following one hour blocking with LiCor Odyssey Blocking Buffer, the membrane was incubated for one hour in 1:15,000 anti-hIgG 800CW in Odyssey Blocking Buffer. The membrane was then rinsed with PBS and imaged using a LiCor Odyssey-CLx with auto-exposure settings.

### Biolayer interferometry

The OctetRED_384_ (ForteBio Inc) was used for label-free measurements of binding as detected on biosensors as a function of optical thickness (nm) versus time as described in^55^. All assay steps were performed at 25 °C with 1000 RPM stirring in tilted-bottom 384-well plates (ForteBio Inc) containing 80 μL sample volume. PBS (pH 7.4) containing 0.1% Tween 20 and 10 mg/mL bovine serum albumin (BSA) was used as the kinetic assay buffer. For kinetic determination, approximately 0.5 nm optical thickness Fab was loaded onto Protein L biosensors. Next, an association phase was performed over a range of RAD51 concentrations for 2–3 minutes. Biosensors were then moved to kinetics buffer alone to measure the rate of dissociation. Association and dissociation rates and dissociation constants were calculated using ForteBio Data Analysis version 7.1 curve-fitting software with a 1:1 Langmuir binding model. A reference well with buffer alone was subtracted from all values to account for sensor drift.

To calculate the IC_50_ for Fab inhibition of RAD51 DNA binding, 1 nm optical thickness of 5′-biotinylated oligo(dT)_36_ was loaded onto a streptavidin biosensor from a 1 μM solution. Association to 0.5 μM RAD51 in the absence of Fab-F2 was performed to yield maximal binding at equilibrium, from which the relative binding at equilibrium of parallel wells containing Fab-F2 could be subtracted.

### ATPase assay

2 μM RAD51 was incubated at 37 °C with Fab-F2 in 100 μL of 50 mM HEPES buffer (pH 7.4) containing 1 mM MgCl_2_, 45 mM NaCl, 3% glycerol, 0.6 mM 2-mercaptoethanol, 1 mM dithiothreitol, 30 μM EDTA, and 0.1 mg/mL bovine serum albumin (BSA), in the presence of 20 μM oligo(dT)_36_. After 10 minutes, the reaction was initiated by addition of 50 mM ATP. The 20 μl reaction was quenched after 20 minutes with 30 μl of 100 mM EDTA. The release of inorganic phosphate by ATP hydrolysis was measured with the malachite green assay described in^77^ modified from^64^.

### Protein labeling

500 ug of Fab or anti-IgG antibody was was dissolved in 600 μL of PBS buffer at room temperature and mixed with 1.35 μL of 10 mg/mL IRDye800CW for 2 hours followed by 16 hours overnight at 4 °C in the absence of light. Free IRDye800CW was removed from the protein conjugates using Zeba® Desalting Spin Columns (Thermo Scientific) per manufacturer’s instructions. IRDye800CW/Fab ratio and Fab concentration were determined by measuring the absorbance of the conjugate at 280 nm and 780 nm using a UV-Vis spectrophotometer, and calculated per the IRDye 800CW Protein Labeling Kit.

### Cell culture and transfection

Mammalian cell cultures were maintained at 37 °C with 5% CO_2_. HEK293T cells were cultured in Dulbecco’s Modified Eagle’s Medium. Cells were passaged to 10–20% confluency for general maintenance once they reached 80–90%. pcDNA-SCFV-FC was transfected into HEK293T cells using Lipofectamine 2000® Reagent (Invitrogen) per manufacturer’s instructions. Cells were cultured for 48 hours and then collected for experimentation. A portion of the sample was lysed for Western analysis to confirm the presence of scFv-Fc expression.

### Clonogenic survival assay

Clonogenic survival assay was used to test the sensitivity of HEK293T cells to increasing doses of a DNA-damaging agent in the presence of Fab or scFv-Fc^72^. HEK293T cells were trypsinized and reseeded in a 6-well tissue culture plate at 200 cells/well. Following overnight culture, cells were treated with indicated concentrations of MMS alone, or in combination with the indicated amount of Fab-F2 or Fab-F2-iPTD. For the scFv-Fc, cells transfected with pcDNA-SCFV-FC were seeded in a 6-well tissue culture plate at 200 cells/well. Following overnight culture, MMS was added at indicated concentrations. After 7 days, cells were fixed and stained using staining solution (0.3% crystal violet, 50% methanol in PBS). Colonies were counted using light microscopy (EVOS® FL Cell Imaging System, ThermoFisher Scientific). Cells treated with PBS and DMSO, or empty vector transfected cells were used as negative controls.

### Fluorescence imaging and flow cytometry

Fab internalization into HEK293T cells was analyzed using fluorescent microscopy (EVOS® FL Cell Imaging System, Cy7 light box, ThermoFisher Scientific). Cells were seeded in a 48-well plate at 5 × 10^4^ cells/well, and IRDye800CW dye-labeled Fab was added to the culture medium as indicated. For imaging, cells in each well were washed with 300 µL PBS. For quantitative analysis of fluorescent Fab uptake, cells were detached from wells with TripLE Express (ThermoFisher Scientific), washed twice with PBS, and the internalized fluorescence signal was measured using a Gallios Flow Cytometer (Beckman Coulter, Inc.). The IRDye800CW dye was excited with the 640 nm laser and fluorescence emission was monitored using a 755 LP filter. Five thousand cells were measured for each sample. Untreated cells were used to set the gate on live cells and mean fluorescence intensity for the gated cells was reported.

### Statistical analysis

P-values were determined using an unpaired *t* test using SPSS 16.0 (SPSS, USA).

## Discussion

In this study we used phage-display to generate a novel synthetic Fab targeting human RAD51. Fab-F2 bound RAD51 with high-affinity (K_D_ = 8.10 nM) and inhibited RAD51 ssDNA binding *in vitro*. The corresponding intracellular scFv caused growth impairment for HEK293T cells transfected with an scFv-Fc intrabody construct. To enable crossing of the cell membrane, we fused Fab-F2 to a novel cell-penetrating peptide, iPTD. Fab-F2-iPTD was easily purified and retained strong binding (K_D_ = 18.20 nM) to RAD51. As compared with Fab-F2, Fab-F2-iPTD was readily imported into HEK293T cells and exerted an intracellular phenotype related to RAD51 inhibition by enhancing the cell killing of the DNA alkylating agent MMS. To our knowledge, this is the first report of a completely RAD51-specific inhibitory antibody fragment. Furthermore, the iPTD fusion Fab opens the opportunity for its use as a viable companion drug to enhance DNA-damage based chemotherapies, and to help treat cancers prone to chemotherapy resistance via homologous recombination mechanisms.

While our study was in progress, another group reported a cell-penetrating antibody with affinity for human RAD51^78^. They investigated a characteristic autoantibody (3E10) from the autoimmune disease systemic lupus erythematosus, and found that it possesses dual specificity to bind RAD51 in addition to its well-known binding of DNA^79^. Significantly, the anti-DNA component is absolutely required for cell-penetration and 3E10 scFv fragments with DNA-binding mutations were unable to penetrate cells and inhibit RAD51^78^. The authors suggest that 3E10 DNA binding is non-toxic, however, it remains to be seen how its connection to a human disease may lead to success in clinical trials. The K_D_ for 3E10 binding RAD51 was 388 nM, and 612 nM for a corresponding scFv antibody fragment, which is over 20-fold weaker as compared with the affinity of Fab-F2-iPTD for RAD51 (18.2 nM) reported in this study. Despite these limitations, 3E10 has attracted interest in clinical development and underscores the significance of our Fab-F2-iPTD.

Cell penetrating peptides are of growing therapeutic interest as they not only enable cellular import, but also facilitate cargo delivery into difficult to penetrate tissues such as tumors, and even allow crossing of the blood-brain barrier^73,80^. Well over 1000 unique CPPs have been experimentally tested to date^81^, and have been used to transport a wide variety of drugs and macromolecules including proteins, nucleic acids, and lipids^82,83^. However, relatively few CPPs reported to date have been used for antibodies and antibody fragments (e.g.)^84–86^. One reason may be because most CPPs are chemically conjugated and site-directed modifications are more difficult with large antibodies versus small molecules, leading to undesired functional compromises. For example, a study using an scFv chemically coupled to a well-characterized TAT CPP^87^ led to severely reduced tumor targeting performance as compared to the corresponding unconjugated antibody^86^. Our iPTD CPP is a genetic fusion and is therefore translated along with the antibody fragment in a 1:1 site-specific manner. We expect the iPTD will be useful for other antibody fragments selected from our synthetic library based on the 4D5 human IgG_1_ Fab framework, and may also be successful with other antibodies sharing the same scaffold^88^.

Our overall goal was to create a therapeutic antibody that inhibits the intracellular protein RAD51. To demonstrate that F2 antibody fragments have the potential to disrupt RAD51 function, we first tested an intracellular antibody based on Fab-F2 and found a strong growth inhibitory phenotype in the absence of exogenous DNA damage treatment. We suspect this phenotype is associated with the more recently identified role for RAD51 in protecting nascent DNA strands during replication^22,23,89^. The phenotype was not observed with treatments using purified Fab-F2 or Fab-F2-iPTD, possibly due to the transient nature of these treatments as compared with constitutive expression of the intrabody and/or the presence of an NLS in the transfected construct. Nonetheless, the phenotype exerted by the scFv-Fc expression construct could be a valuable research tool to study DNA damage-independent processes of RAD51 by reverse genetics. Such reagents are especially useful because *RAD51* deletion mutants cause embryonic lethality^6,90^.

## Supplementary information

Supplementary Information

## Data Availability

The datasets generated during and/or analysed during the current study are available from the corresponding author on reasonable request.
