# Supplementary material for: A Novel Cell-Penetrating Antibody Fragment Inhibits the DNA Repair Protein RAD51
Source: Sci Rep. 2019 Aug 2;9:11227. doi: 10.1038/s41598-019-47600-y (PMC6677837; doi:10.1038/s41598-019-47600-y)
Supplement: Supplementary file 1 — Supplementary Information [file 41598_2019_47600_MOESM1_ESM.docx]

**Supplementary information**

**A Novel Cell-Penetrating Antibody Fragment Inhibits the DNA Repair Protein RAD51**

Landon Pastushok^1,3,+^, Yongpeng Fu^1,+^, Leo Lin^4^, Yu Luo^2^, John DeCoteau^1,3^, Ken Lee^4^, C. Ronald Geyer^1,3,*^

^1^ Department of Pathology and Lab Medicine, University of Saskatchewan, Saskatoon, Canada

^2^ Department of Biochemistry, University of Saskatchewan, Saskatoon, Canada

^3^ Advanced Diagnostics Research Lab, Saskatchewan Cancer Agency, Canada

^4^ iProgen Biotech Inc., British Columbia, Canada

* corresponding author

+ these authors contributed equally to the work

**Corresponding author:**

C. Ronald Geyer

Department of Pathology and Laboratory Medicine,

University of Saskatchewan,

Saskatoon, SK, Canada, S7N 5E5

ron.geyer@usask.ca

**S1**


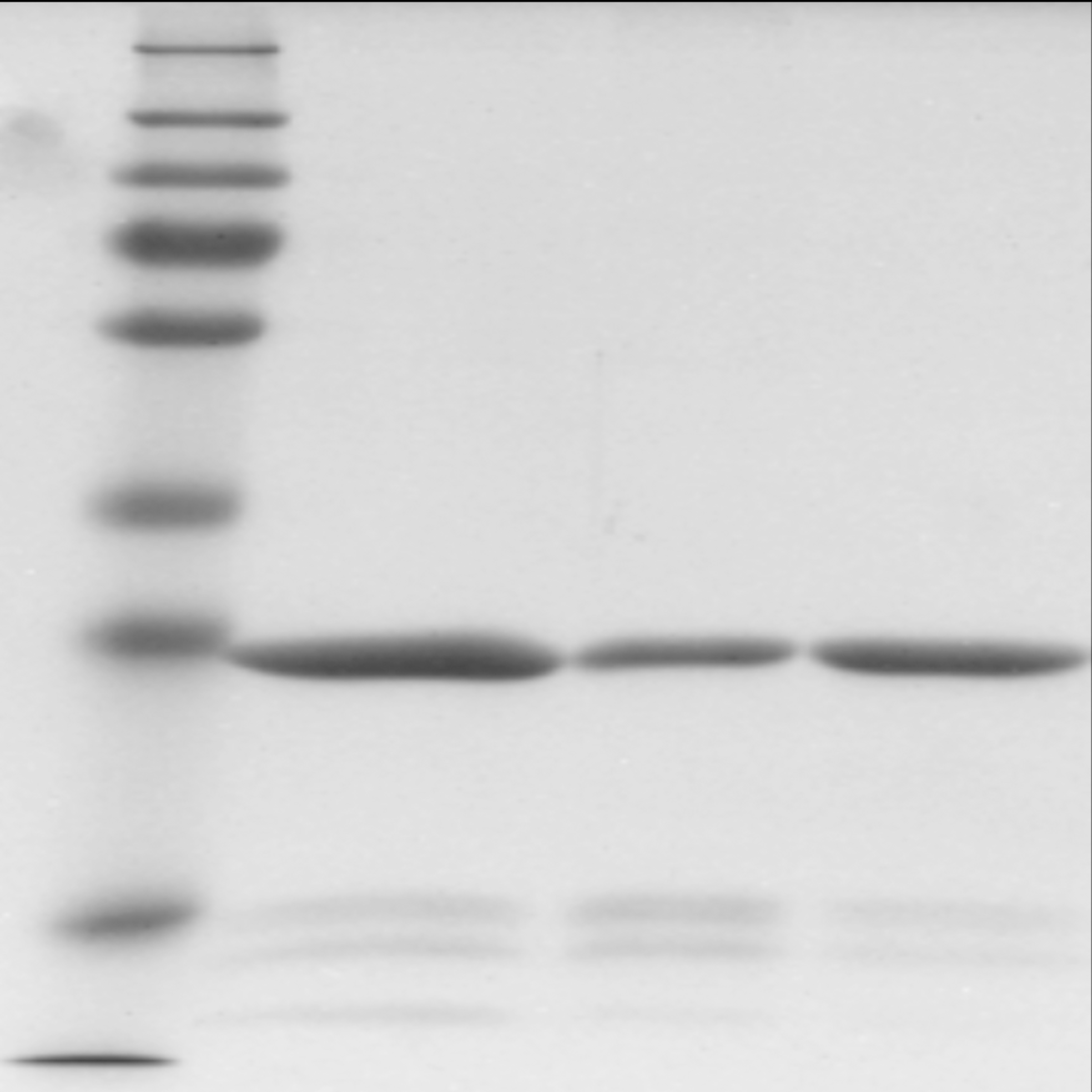


70 kD

35 kD

55 kD

15 kD

10 kD

Fab-F3

Fab-F2

Fab-F1

25 kD


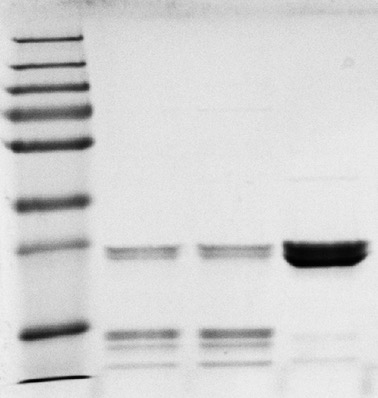


25 kD

55 kD

70 kD

10 kD

Fab-F2-iPTD

35 kD

15 kD

Figure S1 - Fab proteins are purified to near homogeneity.

Coomassie-stained SDS-PAGE shows that Fab-F2 (upper) and Fab-F2-iPTD (lower) are purified to near homogeneity. The iPTD sequence that was added to the heavy chain in Fab-F2-iPTD generates the doublet not seen in the untagged form.

**S2**


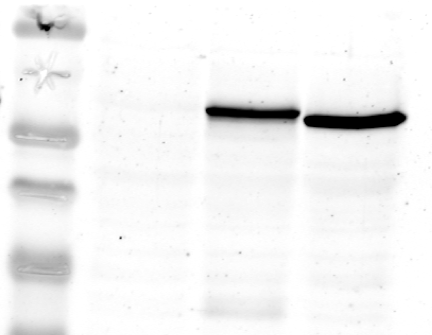


55 kD

pcDNA

pcDNA-SCFV-

FC-F2

70 kD

Figure S2 - Expression of scFv-Fc-F2 protein in HEK293T cells.

HEK293T cells were transfected with pcDNA-SCFV-FC-F2 plasmid and a Western blot using anti-IgG antibody was used to detect expression of the scFv-Fc-F2 antibody fragment.


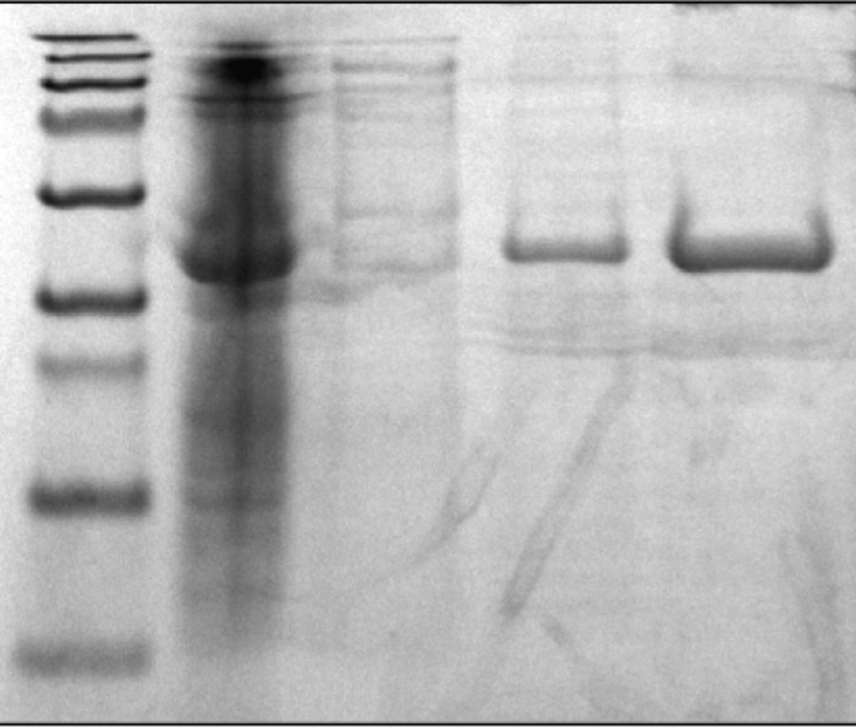


55 kD
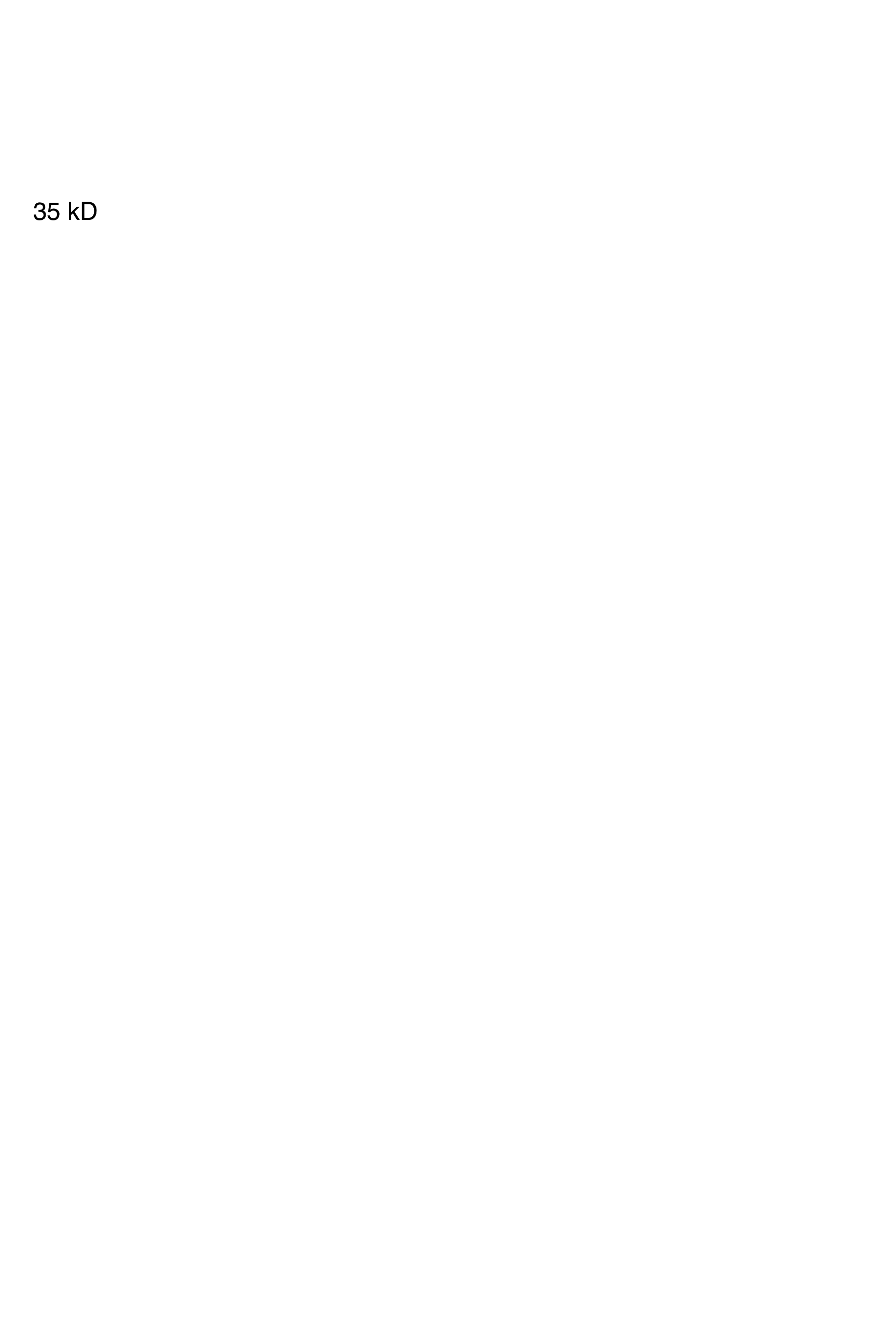

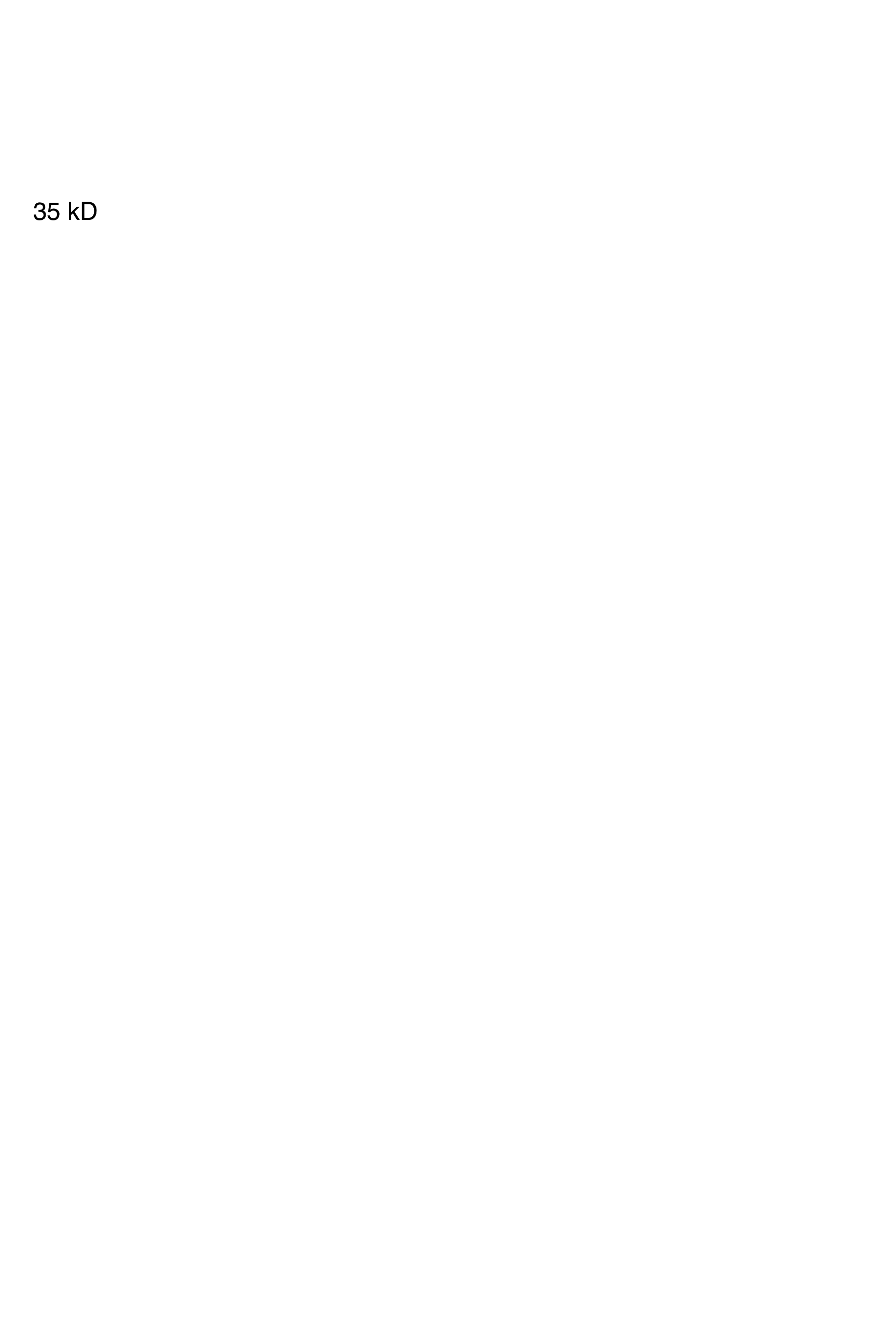


70 kD
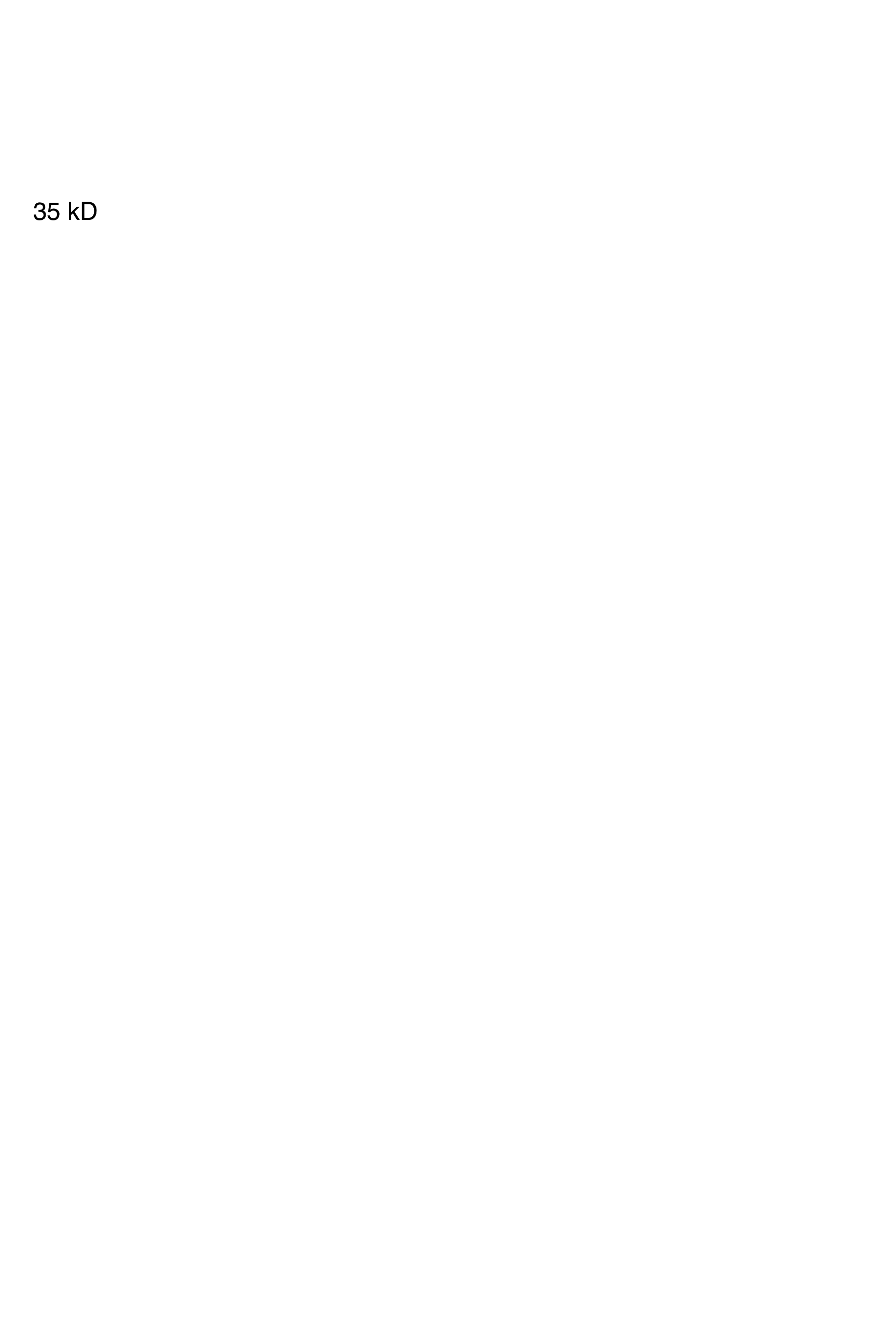

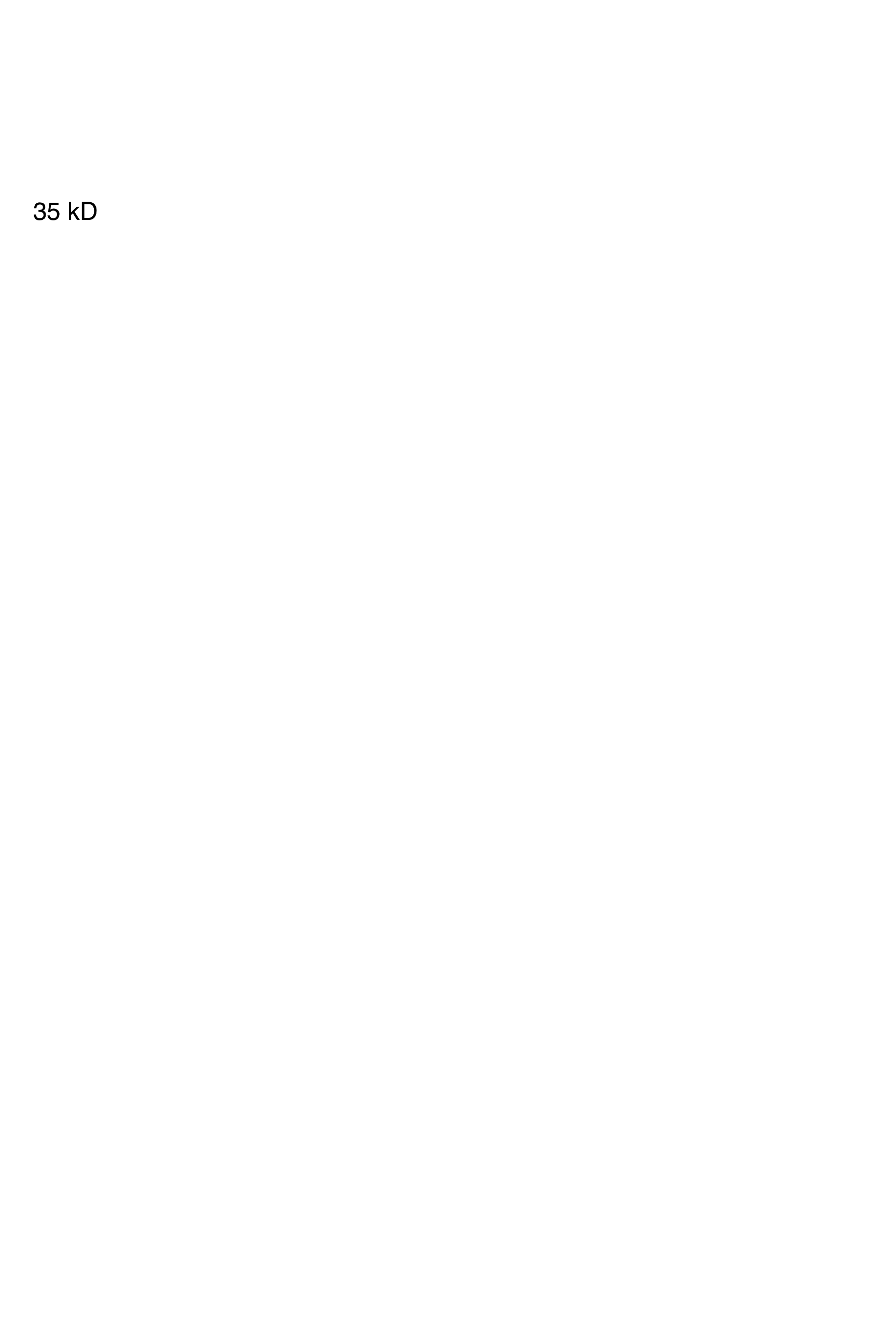


15 kD

10 kD

35 kD

25 kD

Purified RAD51

Figure S3 - RAD51 protein purified to near homogeneity.

Coomassie-stained SDS-PAGE shows that RAD51 was purified to near homogeneity (right-most lane).


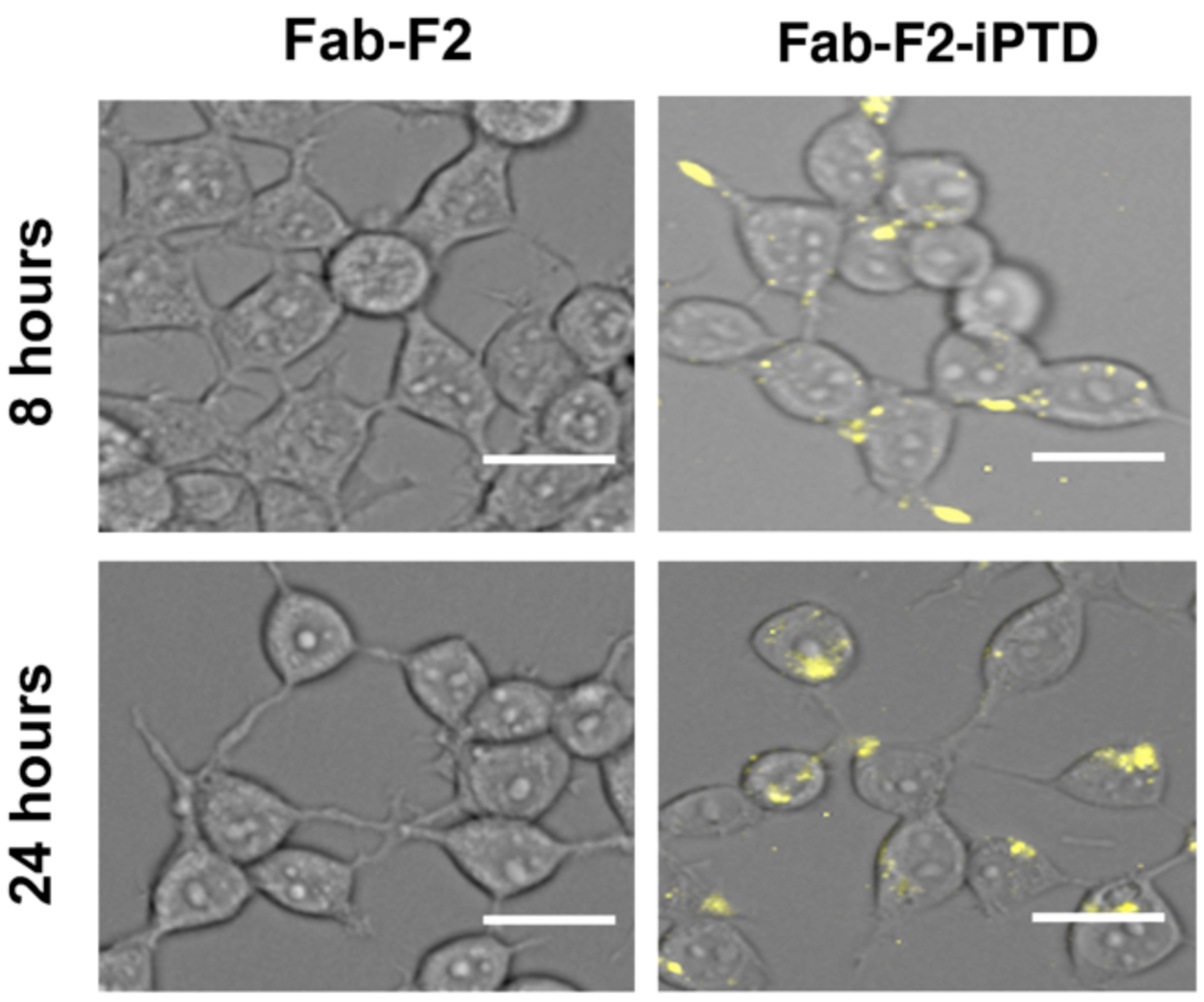


Figure S4 - Internalization of Fab-F2-iPTD into HEK293T cells.

Shown is a zoomed-in levels-adjusted excerpt of panels from Figure 5B to more clearly demonstrate localization of Fab-F2 and Fab-F2-iPTD. HEK293T cells were incubated with 40 µM 800CW-labeled Fab (yellow) for the indicated time points and fluorescent microscopy was used to visualize cellular localization. Image contrast and brightness adjustments were performed equally across all panels. Bars indicate 200 µm.
